# Hand Sanitiser Provision for Reducing Illness Absences in Primary School Children: A Cluster Randomised Trial

**DOI:** 10.1371/journal.pmed.1001700

**Published:** 2014-08-12

**Authors:** Patricia Priest, Joanne E. McKenzie, Rick Audas, Marion Poore, Cheryl Brunton, Lesley Reeves

**Affiliations:** 1 Department of Preventive and Social Medicine, Dunedin School of Medicine, University of Otago, Dunedin, New Zealand; 2 School of Public Health and Preventive Medicine, Monash University, Melbourne Victoria, Australia; 3 Faculty of Medicine, Memorial University of Newfoundland, St John's, Newfoundland, Canada; 4 Public Health South, Southern District Health Board, Dunedin, New Zealand; 5 Community and Public Health, Canterbury District Health Board, Christchurch, New Zealand; Stanford University, United States of America

## Abstract

**Background:**

The potential for transmission of infectious diseases offered by the school environment are likely to be an important contributor to the rates of infectious disease experienced by children. This study aimed to test whether the addition of hand sanitiser in primary school classrooms compared with usual hand hygiene would reduce illness absences in primary school children in New Zealand.

**Methods and Findings:**

This parallel-group cluster randomised trial took place in 68 primary schools, where schools were allocated using restricted randomisation (1∶1 ratio) to the intervention or control group. All children (aged 5 to 11 y) in attendance at participating schools received an in-class hand hygiene education session. Schools in the intervention group were provided with alcohol-based hand sanitiser dispensers in classrooms for the winter school terms (27 April to 25 September 2009). Control schools received only the hand hygiene education session. The primary outcome was the number of absence episodes due to any illness among 2,443 follow-up children whose caregivers were telephoned after each absence from school. Secondary outcomes measured among follow-up children were the number of absence episodes due to specific illness (respiratory or gastrointestinal), length of illness and illness absence episodes, and number of episodes where at least one other member of the household became ill subsequently (child or adult). We also examined whether provision of sanitiser was associated with experience of a skin reaction. The number of absences for any reason and the length of the absence episode were measured in all primary school children enrolled at the schools. Children, school administrative staff, and the school liaison research assistants were not blind to group allocation. Outcome assessors of follow-up children were blind to group allocation. Of the 1,301 and 1,142 follow-up children in the hand sanitiser and control groups, respectively, the rate of absence episodes due to illness per 100 child-days was similar (1.21 and 1.16, respectively, incidence rate ratio 1.06, 95% CI 0.94 to 1.18). The provision of an alcohol-based hand sanitiser dispenser in classrooms was not effective in reducing rates of absence episodes due to respiratory or gastrointestinal illness, the length of illness or illness absence episodes, or the rate of subsequent infection for other members of the household in these children. The percentage of children experiencing a skin reaction was similar (10.4% hand sanitiser versus 10.3% control, risk ratio 1.01, 95% CI 0.78 to 1.30). The rate or length of absence episodes for any reason measured for all children also did not differ between groups. Limitations of the study include that the study was conducted during an influenza pandemic, with associated public health messaging about hand hygiene, which may have increased hand hygiene among all children and thereby reduced any additional effectiveness of sanitiser provision. We did not quite achieve the planned sample size of 1,350 follow-up children per group, although we still obtained precise estimates of the intervention effects. Also, it is possible that follow-up children were healthier than non-participating eligible children, with therefore less to gain from improved hand hygiene. However, lack of effectiveness of hand sanitiser provision on the rate of absences among all children suggests that this may not be the explanation.

**Conclusions:**

The provision of hand sanitiser in addition to usual hand hygiene in primary schools in New Zealand did not prevent disease of severity sufficient to cause school absence.

**Trial registration:**

Australian New Zealand Clinical Trials Registry ACTRN12609000478213

*Please see later in the article for the Editors' Summary*

## Abstract 2

In a cluster randomized trial, Patricia Priest and colleagues find that providing hand sanitizer along with hand hygiene education in primary school classrooms, compared with hand hygiene alone, does not reduce school absences.

*Please see later in the article for the Editors' Summary*

## Editors' Summary

**Background:**

Throughout human history, infectious diseases have been major killers. In the 1300 s, for example, the black death killed a third of the European population. Other diseases such as smallpox and cholera have also devastated human populations. Now, though, a better understanding of the bacteria, viruses, and other microbes that cause infectious diseases and the availability of effective vaccines and antibiotics mean that, for the first time in human history, non-communicable (chronic) diseases such as heart attacks and strokes are killing and disabling more people around the world than infectious diseases. But this does not mean that we can be complacent about infectious diseases. The control of infectious diseases remains important, even in high-income countries, because of the contribution of infectious diseases to ill-health and because we need to manage the risk of epidemics and pandemics (disease outbreaks that affect a large proportion of the population of a country or the world, respectively) of influenza and other diseases.

**Why Was This Study Done?:**

The control of infectious disease transmission in children is a particularly important component of disease control because children tend to have high rates of infectious disease and to have more physical contact with peers and with adults than other age groups, particularly in the school environment. It might be possible, therefore, to reduce the occurrence of many infectious respiratory and gastrointestinal diseases in communities by interrupting the transmission of infectious diseases between children at school, but how can this be achieved? In health care settings, good hand hygiene is a key component of infectious disease control, so, here, the researchers undertake a cluster randomized trial among primary school children in New Zealand to investigate whether the promotion of extra hand cleaning through the provision of alcohol-based hand sanitizer in classrooms can reduce illness absences among school children compared with normal hand hygiene (washing with soap and water, mainly in school bathrooms). A cluster randomized trial compares the outcomes of groups of participants (in this case, schools) chosen randomly to receive different interventions.

**What Did the Researchers Do and Find?:**

The researchers randomly assigned 68 city primary schools to the intervention or control group. All the children (aged 5–11 years) attending the participating schools received a thirty-minute in-class hand hygiene education session. Alcohol-based hand sanitizer dispensers were installed in the classrooms of the intervention schools during the winter term, and the children were asked to use the dispensers after coughing or sneezing and on the way out of the classroom for morning break and lunch. The researchers report that the trial's primary outcome—the rate of absence episodes per 100 child-days due to any illness among “follow-up” children, individuals whose caregivers agreed to be asked about the reason for any absence—was similar in the intervention and control groups. Moreover, among the follow-up children, the provision of hand sanitizer did not reduce the number of absences due to a specific illness (respiratory or gastrointestinal), the length of illness and length of absence from school, or the number of episodes in which at least one other family member became ill. Finally, the number of absences for any reason, and length of absence episodes, in all the children enrolled at the participating schools did not differ between the intervention and control groups.

**What Do These Findings Mean?:**

These findings suggest that the provision of hand sanitizer in addition to usual hand hygiene in primary schools in New Zealand did not prevent any infectious diseases severe enough to warrant school absence. Because the trial was undertaken during an influenza epidemic, influenza-related public health messages about good hand hygiene may have increased hand hygiene among all the children in the study and lessened the intervention's effectiveness. Other study limitations—including that only a third of caregivers agreed to be contacted about their child's absences, and these may have been caregivers who had already taught their children good hand hygiene—may also affect the accuracy of these findings and their generalizability to other high-income countries. However, these findings suggest that, in high-income countries where clean water for hand washing is readily available, putting resources into extra hand hygiene by providing hand sanitizer in classrooms may not be an effective way to break the child-to-child transmission of infectious diseases.

**Additional Information:**

Please access these websites via the online version of this summary at http://dx.doi.org/10.1371/journal.pmed.1001700.

- The US Centers for Disease Control and Prevention has information about hand-washing, when and how to wash your hands and use sanitizer, and hand-washing as a family activity; it also provides information about the importance of hand hygiene in health care settings
- Public Health England provides information about hand-washing; its webpage about hand-washing in primary schools contains links to lesson plans about hand-washing for children aged 5–7 years and to e-Bug, a web-based student resource about infectious diseases and their prevention for children aged 7–14 years
- Kidshealth, a US-based not-for-profit organization, also provides information about the importance of hand-washing for parents, kids, and teens (in English and Spanish)

## Introduction

While the global impact of infectious diseases on death and disability is outweighed by that of non-communicable disease [1], infectious diseases continue to cause ill health in high-income countries [2], and management of the risk of influenza or other pandemics remains important. Children are important in community disease transmission [3] because they have high rates of infectious disease and tend to have more physical contact with peers and adults than other age groups. In addition, when children are unable to attend school because of illness, family members may have to stay home from work to care for them [3]. Therefore, reducing infectious disease among children has the potential for wide-ranging benefits to society.

Reduction of the incidence of many infectious respiratory and gastrointestinal diseases requires interruption of person-to-person transmission, and the school environment is likely to be an important setting for the transmission of infectious diseases because children are in close contact over long periods. Given that it is not feasible to require all infectious children to stay away from school (because of asymptomatic carriage, high incidence, and often mild symptoms), reduction of transmission between children at school could be an effective way of reducing the incidence of infectious disease among children. Hand hygiene is recognised as a key measure to reduce infectious disease transmission in health care settings [4], and interventions that aim to improve hand hygiene compared with usual practice have been shown to reduce infectious disease risk in the community [5], with typically larger effects observed for gastrointestinal [6] than for respiratory [7] diseases. The study settings have varied, but a number of studies have been conducted in schools [8]–[14]. A review of school-based studies noted that they have generally found beneficial effects of hand hygiene interventions; however, reporting of the methodological characteristics of these studies (which may affect the validity of the results) was generally poor, and the analyses were generally inappropriate (without adjustment for the clustered nature of the design) [15]. Additionally, there is a dearth of large pragmatic trials [7]. A range of different hand hygiene interventions have been tested in primary schools, often in combination with hand-washing education, including mandatory hand-washing [12], providing soap for school basins [8], and providing hand sanitiser [9]–[11],[13],[14]. Studies of providing hand sanitiser in schools have tended to be small or to involve rather intense interventions (e.g., daily wiping of all classroom surfaces at lunchtime [13]) that may not be widely feasible.

We aimed to test whether the addition of hand sanitiser in primary school classrooms compared with usual hand hygiene (use of soap and water, mainly in school bathrooms) would reduce illness absences in primary school children in New Zealand. We chose to use hand sanitiser as our hand hygiene modality in preference to increasing use of existing facilities because school bathroom hand-washing facilities are of variable quality [16],[17], which might be a barrier to attempts to increase hand-washing. Improving and maintaining bathroom facilities in a large number of schools would be a major undertaking, and hand sanitiser is an acceptable alternative to hand-washing with soap and water for children [18]. We provided hand sanitiser in classrooms rather than in the bathroom facilities to promote extra hand cleaning in additional to that usually undertaken, and to ensure that use was largely supervised.

### Objectives

Our primary objective was to assess whether the provision of hand sanitiser in primary school classrooms in the South Island of New Zealand reduced the incidence rate of absence episodes due to any illness in children, during the winter terms.

Secondary objectives included assessing whether hand sanitiser was effective in reducing the (i) incidence rate of respiratory illness absence episodes, (ii) incidence rate of gastrointestinal illness absence episodes, (iii) incidence rate of absence for any reason, (iv) length of illness episode, (v) length of illness absence episode, and (vi) incidence rate of subsequent illness among other children or adults in the household. We also examined whether the use of hand sanitiser was associated with an increased risk of any skin reactions during the intervention period.

## Methods

The protocol for this trial has been published [19], and a brief description of the methods follows.

### Ethics Statement

The New Zealand Multi-Region Health and Disability Ethics Committee provided approval for the trial on 13 March 2009 (MEC/09/01/005). School principals gave permission for their school to take part in the study. Parents/guardians (henceforth “caregivers”) of follow-up children gave written consent to be telephoned following their children's absences from school.

### Setting and Participants

The study took place in the three cities within the regions covered by the New Zealand Ministry of Health Public Health Units of investigators (M. P. and C. B.). Only city schools were eligible for inclusion because of the increased cost associated with weekly visits to schools more widely distributed within the regions.

We evaluated the effectiveness of the intervention over the winter school terms (27 April to 25 September 2009) by measuring the absence rates of all children enrolled at the participating schools at any time during the study period, and by collecting more detailed information on the absences of a subgroup of these children, the follow-up group, whose caregivers were contacted via telephone when the child had been absent from school. We aimed to recruit 50 children from each school into the follow-up group. At the beginning of March 2009, letters inviting participation in the study (including a consent form and baseline questionnaire) were distributed by the school to students who had been randomly selected from the school roll.

All eligible schools were invited to take part in the study. The study sample comprised primary schools that met the inclusion criteria below and whose principal consented for the school to participate in the trial, be randomised, and potentially receive the hand sanitiser.

### Inclusion Criteria

All schools (i) with at least 100 children in school years one to six (aged 5 to 11 y) enrolled at the school in November 2008; (ii) located within the city boundaries of Christchurch, Dunedin, or Invercargill, in the South Island of New Zealand; (iii) not “special schools” (e.g., schools for children with deafness or disability); and (iv) either not currently using hand sanitiser products or willing to not use hand sanitiser products for the period of the trial if they were randomised to the control group were eligible to participate in the trial.

Children were eligible to participate in the follow-up group, for whom more detailed information on absences was collected, if they attended a school year 1 to 6 class in one of the included schools at the beginning of the second school term in 2009 (the end of April), and their caregivers completed the consent form indicating that they were willing to be telephoned following their child's absences and that they were able to take part in telephone interviews in English.

### Exclusion Criteria

Potentially eligible follow-up children were excluded if a caregiver was an investigator or study personnel of the trial, or if the principal of the school directed us not to approach their family.

### Intervention

Children in intervention schools received an approximately 30-min in-class hand hygiene education session and were also instructed in the use of hand sanitiser and asked to use it after coughing/sneezing, and on the way out of the classroom for morning break and for lunch. Caregivers of children at intervention schools were sent a letter home with the school newsletter explaining the study and asking them to let their child's teacher know if they did not wish their child to use the hand sanitiser, or if during the study they wished their child to stop using the sanitiser for any reason.

During the school holidays in April 2009, “no touch” dispensers, which dispensed approximately 0.45-ml of alcohol-based sanitiser (>60% ethanol) when hands were placed under an infrared sensor, were fitted in all classrooms in intervention schools. School liaison research assistants subsequently visited each classroom weekly to top up the sanitiser during the course of the study, which continued from 27 April to 25 September 2009 (20 school weeks). The quantity used in each classroom was recorded.

### Control

Children in the control schools received the same in-class hand hygiene education sessions as the intervention schools (minus the instructions on classroom hand sanitiser use), to ensure that the children in the two study groups were equivalent in their exposure to hand hygiene education prior to the study.

### Outcome

Table 1 shows the outcome measures that were planned and collected. The primary outcome was the number of absence episodes due to any illness among follow-up children.

**Table 1:** Planned outcomes from protocol [19] and outcomes actually measured.

Outcome	Planned	Collected	Definition
**Primary outcome**			
Number of absence episodes due to any illness^1^	Y	Y	An absence episode that, in the follow-up phone call, was reported to be due to any illness. An absence episode was defined as a series of one or more days of absence from school, with a new episode defined as one in which there were at least three days with no absence since the previous absence episode (including week and weekend days).
**Secondary outcomes in follow-up children**			
Number of absence episodes due to respiratory illness	Y^2^	Y	An absence episode due to illness that includes at least two of the following caregiver-reported symptoms for 1 d, or one of the following symptoms for 2 d (but not fever alone): runny nose, stuffy or blocked nose or noisy breathing, cough, fever, sore throat or sneezing.
Number of absence episodes due to gastrointestinal illness	Y	Y	An absence episode due to illness that does not meet the criteria for respiratory illness and includes either diarrhoea or vomiting or both for at least 1 d.
Length of illness absence episode	Y	Y	Number of days the child was absent from school during an illness episode.
Length of illness episode	Y^2^	Y	Number of days the illness episode lasted; calculated as the number of days from the first to last day of the absence episode, plus 1 d if the first day of the absence episode was a Monday and plus 1 d if the last day of the absence episode was a Friday.
Number of household members who became ill within 1 wk of the participating child's illness onset	Y	N	
Number of episodes where at least one other adult in the household had the same illness after the child	N	Y	As reported by caregiver in follow-up phone call.
Number of episodes where at least one other child in the household had the same illness after the child	N	Y	As reported by caregiver in follow-up phone call.
**Secondary outcomes in all children**			
Number of absence episodes for any reason	Y	Y	Absence episodes for any reason; identified from the school roll. A new absence is defined in the same way as for the primary outcome.
Length of absence episode for any reason	Y	Y	Number of days the child was absent from school during an absence episode.
**Adverse events**			
Skin reactions^1^	Y	Y	As reported by caregiver in phone call after the end of the study (asked whether child had any skin problems in the winter school terms, and whether any eczema was better, worse, or the same as usual during the study period). A skin reaction was coded as yes if there were any skin problems or if their eczema was worse.

1 Follow-up children only.

2 See Table 2 for detail of change to definition.

### Sample Size

The primary outcome was the number of absence episodes due to illness. Monitoring by the Public Health Unit of absences among primary school children in Dunedin and Invercargill in 2006 and 2007 found an average of 11 absences reported as due to illness per 100 pupil-weeks, equivalent to 2.2 absences per pupil over 20 wk (M. Poore, personal communication). The trial was powered at 80% to detect a 20% reduction in the incidence rate of absence episodes due to illness (from 2.2 to 1.76 episodes per pupil over 20 wk) [5]. Assuming recruitment of 50 follow-up children per school and an intra-cluster correlation of 0.15, 27 schools (1,350 follow-up children) per group would be sufficient to detect a 20% reduction in rates with 80% power (two-sided significance level of 5%). Allowing for 20% attrition in the participation of schools, we aimed to recruit 34 schools per group.

### Randomisation and Allocation Concealment

Schools were randomly allocated to either the hand sanitiser or control group using restricted randomisation. Three strata were defined by geographical area (cities of Christchurch, Dunedin, or Invercargill), and within each stratum schools were randomly allocated with equal probability (1∶1 randomisation ratio) to the hand sanitiser or control group (i.e., 34 schools per group). City was chosen as a stratification variable because outbreaks of gastrointestinal illness or flu may be restricted to an area, and could therefore confound the estimated intervention effect if the intervention groups were not equally distributed within cities.

The study statistician (J. E. M.) was provided with only a numeric school code and its area, and randomised schools to “A” or “B” using random numbers generated by Stata/MP version 10.1 for Windows (StataCorp). Independently, and prior to receiving the allocation list, P. P. randomly allocated “A” and “B” to intervention or control. Randomisation of all schools was undertaken at one time, and the randomisation list was held by P. P. until analysis was complete.

### Blinding

Due to the nature of the intervention, it was not possible to blind the children, school administrative staff, or the school liaison research assistants. The investigators not involved in running the trial (J. E. M., R. A., M. P., and C. B.), the telephone interviewers (outcome assessors), and the statistician (J. E. M.) were blinded to the group allocation until after the analysis was complete.

### Data Collection

The baseline questionnaire sent to the caregivers of follow-up children collected self-reported information on household composition, socio-demographic variables (ethnicity, education, occupation, income, ages of children), paid and unpaid work, family health, and hand hygiene practices.

During the study period, school liaison research assistants visited all schools weekly and collected absence information from the school's records for the previous week for all children in the school. When a follow-up child had been absent and the reason for the absence was recorded as “medical”, “illness”, or “unknown”, the caregiver of the child was telephoned. Contact occurred approximately 9 d after the absence, and the caregiver was asked about the reason for the absence. In cases of illness, further questions were asked about the child's symptoms and their duration, whether others in the family had had the same symptoms, how the child had been cared for during the absence, and the cost of any health care sought because of the illness.

Schools provided information on the total number of children enrolled at the beginning of the study, halfway through the study (when there was a holiday), and at the end of the study period. At the end of the study, the total amount of hand sanitiser used by each classroom in the intervention schools was measured. In addition, we attempted to contact each follow-up child's caregiver, irrespective of whether the child had been absent, to ask about possible adverse effects (skin reactions).

### Analyses

Estimates of intervention effectiveness (incidence rate ratios [IRRs] or odds ratios [ORs]) for follow-up children were calculated from marginal models using generalised estimating equations (GEEs), with robust variance estimation, to account for correlation of responses of children within schools. An exchangeable correlation structure was specified, whereby responses from the same school were assumed to be equally correlated. If the estimated intra-cluster correlation (ICC) from the GEE was negative, the model was refitted assuming an independent correlation structure, i.e., an ICC of zero. This approach yields conservative estimates of standard errors and follows the recommendations of others in assuming that in this context, the likely explanation for a negative ICC is sampling variability and not a true negative ICC [20],[21]. Confidence limits for ICCs were calculated through bootstrapping, using a combination of the *bootstrap* and *xtgee* commands in Stata. Bootstrapping allowed for clustering of responses within schools. Bias-corrected 95% bootstrap confidence intervals were calculated from 5,000 replicates.

Models were adjusted for the stratification variable, which represented city (Christchurch, Dunedin, or Invercargill). As part of the pre-specified secondary analyses, we also fitted models adjusting for the potential confounder school-level deprivation (in addition to city). School-level deprivation reflects the proportion of students who live in more or less advantaged communities, using information from the census on household income, occupation, household crowding, educational qualifications, and income support [22].

For the primary outcome (number of absence episodes due to any illness) we undertook a pre-specified per-protocol analysis that included only intervention and control schools that complied with the protocol. Protocol compliance for the intervention schools was defined as dispensing a volume equivalent to at least 45 ml per child of hand sanitiser solution over the trial period, and for the control schools, not introducing hand sanitisers. Marginal logistic and negative binomial regression models were employed for binary and count outcomes, respectively. The negative binomial heterogeneity parameter used in the marginal models was first estimated from fitting a generalised negative binomial model.

Estimates of the intervention effectiveness for all children were calculated from models fitted on data aggregated to the level of the school. We were unable to analyse absence data from the school rolls at the level of the child, since we could not uniquely identify children (e.g., when they changed class rooms). Negative binomial regression was used to estimate the effectiveness of the intervention, adjusting for the stratification and confounding variables described above.

No adjustment for multiple testing was undertaken. All models were fitted using the statistical package Stata version 12 (StataCorp).

### Deviations from Published Protocol

Some changes were made to the protocol following its publication [19]. All changes were made prior to the commencement of analysis and are summarised in Table 2 (a detailed explanation is available in Deviations S1).

**Table 2:** Summary of protocol deviations.

Original Protocol^1^	What Was Actually Done
**Intervention**	
Both intervention and control schools “will have an in-class session, led by the school liaison research assistant, to discuss hand hygiene.…The purpose of this session is to ensure that the two groups are equivalent with respect to hand hygiene knowledge (or at least having had the opportunity to acquire hand hygiene knowledge) at the beginning of the study.” It was intended that this would be the only hand hygiene education that pupils received during the study period.	The study took place in 2009, during the influenza A(H1N1)pdm09 pandemic. Most schools promoted hand hygiene and other influenza-prevention actions such as covering coughs and sneezes, through encouragement of students and notices in school newsletters.
Inclusion criteria included that “schools are currently not using hand sanitiser products or are willing to not use hand sanitiser products for the period of the trial if they are randomised to the control group.”	In response to public health advice about hand hygiene for pandemic influenza prevention, a few control schools installed hand sanitiser or asked all children to bring their own hand sanitiser to school.
**Outcomes**	
“The length of the illness episode will be defined as the number of days between the first and last day of absence. For children who are absent on only a Monday or only a Friday, we will define the length of the episode as two days.”	The length of the illness episode was calculated to be the number of days from the first to last day of the absence episode, plus one day if the first day of the absence episode was a Monday and plus one day if the last day of the absence episode was a Friday.
“If we are unable to contact the caregivers of the ‘follow-up children’ to ascertain why they were absent, we may be able to determine the reason for the absence from the school administrative staff.”	We found that information about the reason for absence was very variably recorded by schools, so it was decided that information noted on the school rolls would not be used in the analysis of the follow-up children.
“A respiratory illness will be defined as an acute illness that includes at least one of the following symptoms: runny nose, stuffy or blocked nose, cough, fever or chills, sore throat, or sneezing.”	Respiratory illness was defined as an episode of illness that included at least two of the following caregiver-reported symptoms for 1 d or one of these symptoms for 2 d (but not fever alone): runny nose, stuffy or blocked nose or noisy breathing, cough, fever, sore throat, or sneezing.
“A gastrointestinal illness will be defined as an acute illness that includes at least two watery or much looser than normal bowel movements and stools over a 24 hour period and/or vomiting.”	Gastrointestinal illness was defined as an episode of illness that did not meet the criteria for respiratory illness and included either or both of the following symptoms lasting for at least 1 d: diarrhoea or vomiting.
**Analysis**	
“A secondary per-protocol analysis will be undertaken where we will only include schools which complied with their allocated intervention. For the intervention group, we will define schools as complying if they used at least 45 ml per child of hand sanitiser solution over the study period. This usage equates to using the hand sanitiser at least once per day.”	In addition, control schools were defined as complying if they did not install hand sanitisers for use by students at any time throughout the trial.

1 All quotes from protocol [19].

### Trial Registration

The trial was registered with the Australian New Zealand Clinical Trials Registry (ACTRN12609000478213). The trial is recorded as “retrospectively registered”. Many aspects of the intervention (e.g., the specifics of the education session) and data collection processes (e.g., frequency with which the absence data would be collected) were negotiated with the schools to ensure they were acceptable. These negotiations necessarily took place in the school term immediately prior to the roll-out of the intervention. The trial was not registered until these details had been confirmed, which occurred just before the commencement of the trial. Trial registration was first submitted to the registry on 11 March 2009, the same week that we first wrote to caregivers of children asking them whether they would be willing to have a telephone interview following any absences of their children. After some delay the registry asked for some clarification and further details, which were provided, and the trial was registered on 12 June 2009. Data collection had begun on 27 April, but no changes were required by the registry, or made by the investigators, to the methods and measures of the study following the initial registration submission in March.

## Results

### Participants

**Figure 1:**
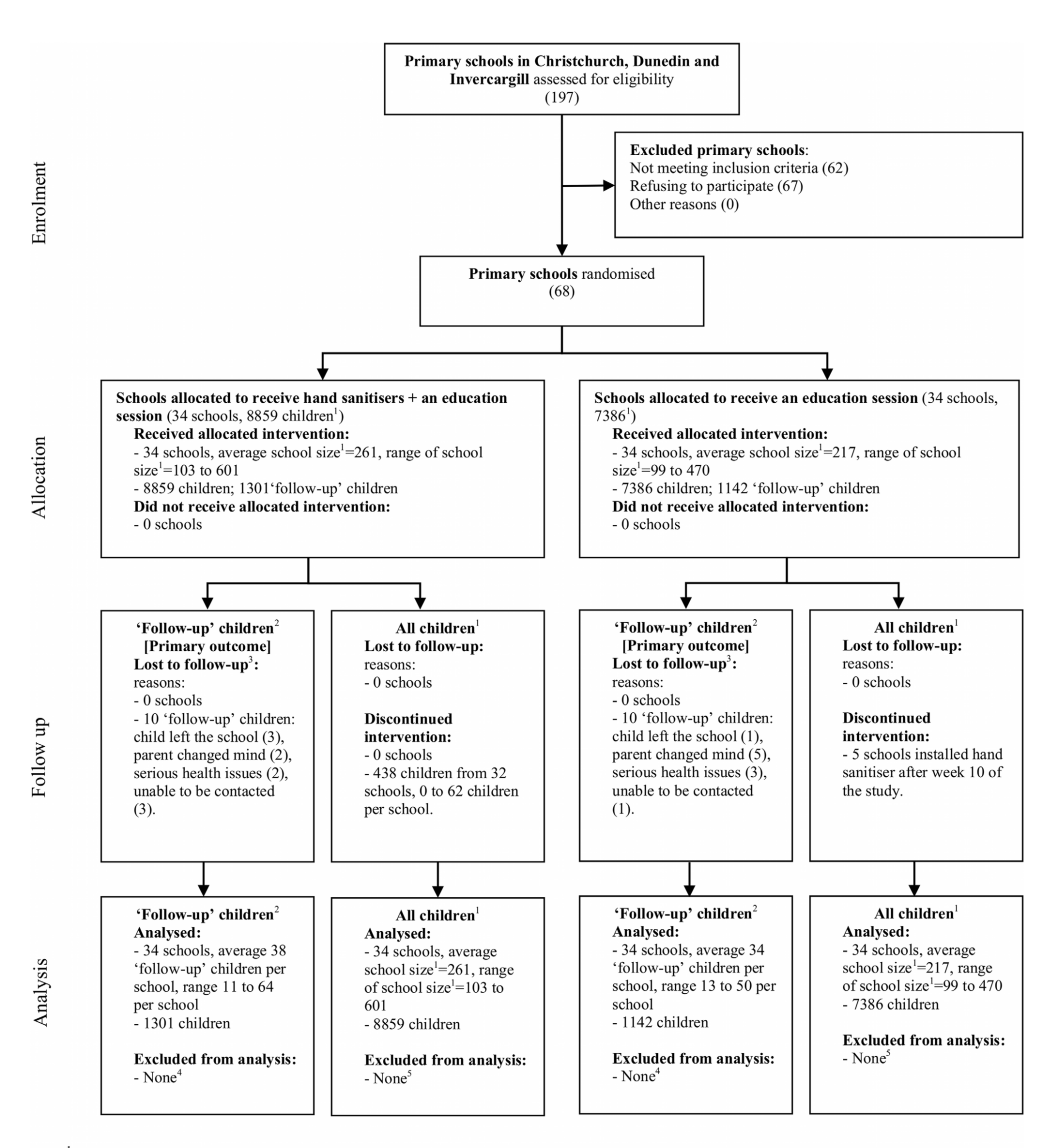
Flow diagram of the progress of schools and children through the trial. ^1^Includes all children in school years 1 to 6 (generally aged from 5 to 11 y). The number given here is the average roll over the period of the trial. ^2^Follow-up children were a randomly selected sample of all children attending the participating schools, whose caregivers were followed up for detailed information about their illness absences. The primary outcome, absence episodes due to illness, is measured only in this group of children. ^3^We may not have been informed about all children who left the schools. ^4^All follow-up children were included in the analysis. The period of time that each child was in the trial (the exposure period) was adjusted for through the statistical model. ^5^All children who had an absence (for any reason) were included in the analysis, even if they were lost to follow-up at some point (e.g., moved schools). The exposure period was calculated as the average of the school roll over the period of the trial (multiplied by 100; the number of school days that were encompassed by the trial period). Figure adapted from [29].

Sixty-eight schools took part in the study; 34 were randomly allocated to each arm of the trial. Figure 1 shows the progress of schools and children through the trial. Participation was higher among smaller schools (64% of schools with a roll size of 100–199 students versus 28% of schools with a roll size of >300 students) and among schools in Dunedin (74%, versus 41% in Christchurch and 50% in Invercargill). Invitations to take part in follow-up telephone interviews if children were absent from school (i.e., follow-up children) were sent to the caregivers of a total of 6,720 children (up to 100 per school; three children were not approached at the instruction of the school principal), and the caregivers of 2,443 (36.4%) children consented. Consent was higher among caregivers of children from schools in Dunedin (40%, versus 35% in Christchurch and 30% in Invercargill) and in less disadvantaged schools (45% in the least disadvantaged schools, versus 19% in the most disadvantaged schools).

### Baseline Characteristics

Caregivers of children in intervention schools were more likely to agree to have their children participate as follow-up children than those in control schools (39% versus 34%). Table 3 summarises the school and follow-up children socio-demographic characteristics. Control schools were less advantaged than intervention schools, but otherwise the groups were well balanced.

**Table 3:** Baseline characteristics.

Category	Characteristic	Hand Sanitiser Group		Control Group	
		Number or Mean	Percent or SD	Number or Mean	Percent or SD
**Schools**	**Total schools**	34		34	
	**Roll just prior to study (mean, SD)**	228.8	115.8	209.6	102.2
	**City**				
	Christchurch	19	55.9%	18	52.9%
	Dunedin	11	32.4%	12	35.3%
	Invercargill	4	11.8%	4	11.8%
	**School decile** *				
	1–3 (least advantaged)	5	14.7%	11	32.4%
	4–7	9	26.5%	6	17.6%
	8–10 (most advantaged)	20	58.8%	17	50.0%
	**Total follow-up children**	1,301		1,142	
**Follow-up children**	**Follow-up children with a baseline questionnaire**	1,287		1,132	
	**Household income (in Australian dollars)**				
	Not stated	101	7.9%	87	7.7%
	$0–$40,000	156	12.1%	163	14.4%
	$40,001–$80,000	525	40.8%	415	36.7%
	$80,001+	505	39.2%	467	41.3%
	**Ethnicity (prioritised)** †				
	Māori	163	12.7%	132	11.7%
	Pacific	31	2.4%	35	3.1%
	Asian	41	3.2%	33	2.9%
	European	1,019	79.2%	907	80.1%
	Other	25	1.9%	21	1.9%
	Not stated	8	0.6%	4	0.4%
	**Education (highest qualification in the household)**				
	No qualification/not stated	51	4.0%	61	5.4%
	Some high school qualification	334	26.0%	309	27.3%
	University	632	49.1%	515	45.5%
	Alternative qualification	270	21.0%	247	21.8%
	**Self-reported overall family hand hygiene**				
	Not stated	82	6.4%	58	5.1%
	Poor/fair	115	8.9%	88	7.8%
	Good/very good/excellent	1,090	84.7%	986	87.1%
	**Household size (mean, SD)**	4.4	1.12	4.36	1.07
	**Number of children in household (mean, SD)**	2.42	0.96	2.43	0.93
	**Children aged under 5 y in household**				
	0	903	70.2%	807	71.3%
	1	322	25.0%	275	24.3%
	2	58	4.5%	48	4.2%
	3	4	0.3%	2	0.2%
	**Caregivers in paid employment**				
	Both caregivers, at least 20 h per week	514	39.9%	455	40.2%
	At least one caregiver employed less than 20 h per week	289	22.5%	219	19.4%
	At least one caregiver not in paid employment	478	37.1%	446	39.4%
	Missing	6	0.5%	12	1.1%

*School-level deprivation uses the decile assigned to each school by the New Zealand Ministry of Education for funding purposes. It reflects the proportion of students who live in more or less advantaged communities, using information from the census on household income, occupation, household crowding, educational qualifications, and income support. Decile 1 schools are in the least advantaged communities, and decile 10 schools in the most advantaged.

† Respondents were asked to tick all the ethnicities represented in their household. Prioritised ethnicity, in New Zealand, codes as Māori participants who report Māori as one of their ethnic groups, as Pacific those who do not report Māori but do report a Pacific ethnicity as one of their ethnic groups, as Asian those who do not report Māori or Pacific ethnicity but report an Asian ethnicity, and the remainder as European (if New Zealand European or another European ethnicity reported) or other (if not).

### Absences

**Figure 2:**
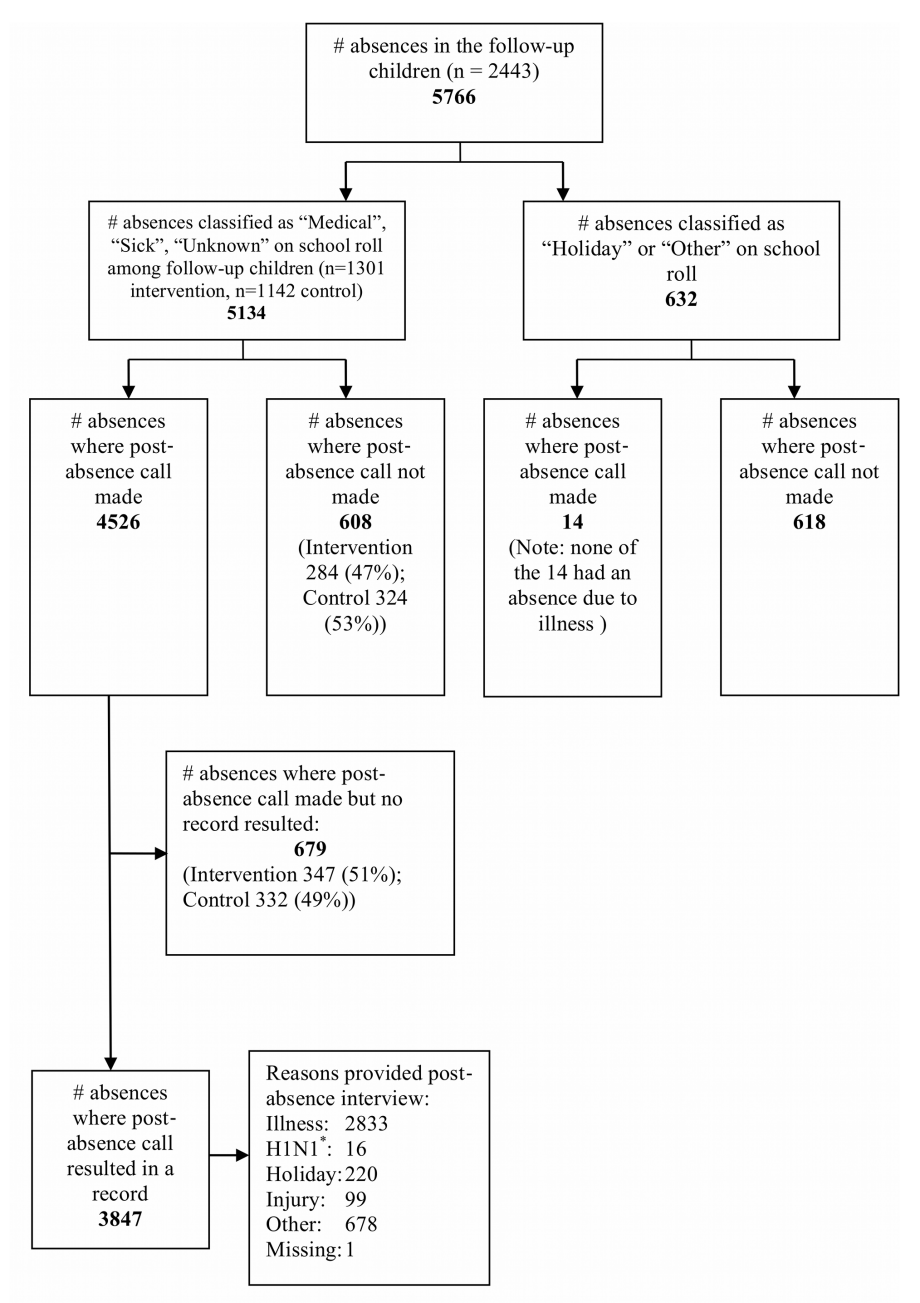
Flow diagram outlining process for identifying the reason for school absences. “H1N1” absences (asterisk) were absences where the child had been asked to stay at home because of possible contact with a known case of H1N1, rather than because they were sick themselves.

Among the 2,443 follow-up children there were 5,766 absences identified from the school rolls, 5,134 of which were recorded as “medical”, “illness”, or “unknown”. Post-absence phone calls resulted in establishing an absence reason for 3,846 absences (74.9%), of which 2,833 were identified as being due to illness (Figure 2). There were 608 absences (13.4%) that should have resulted in a call but did not, 284 in the intervention group and 324 in the control group. Reasons for post-absence calls not being made included errors in identifying that a new absence episode had occurred (i.e., miscounting the gap between the end of one absence episode and the beginning of another) and, for one school in the control group, an administrative error that meant that some follow-up children were not identified as such in the database of absences.

### Intervention Fidelity

A change to the hand sanitiser solution was made in 41 of 396 classrooms (in 9 of 34 schools) halfway through the trial (after 10 wk). Some children had become reluctant to use the initial hand sanitiser product before eating their lunch, because they reported tasting the sanitiser on their fingers and food. We identified a product that did not have a “flavour” and provided that to classes where this complaint had been made. Both brands of sanitiser contained >60% ethanol. Following this change to the product, the median classroom difference in sanitiser usage between the first 10 wk and the second 10 wk among classes that switched products was −220 ml; the inter-quartile range of the usage difference was −420 ml to 645 ml.

All schools in the intervention group complied with the protocol of dispensing a volume equivalent to at least 45 ml per child of hand sanitiser solution over the trial period. The average hand sanitiser solution dispensed per child over the 34 schools was 94 ml (standard deviation = 19). We had not anticipated that control schools would install hand sanitiser, because the inclusion criteria for the study required that they agree not to. However, in view of the pandemic of influenza A(H1N1)pdm09 during the study period, five control schools did install hand sanitiser in response to public health advice. Most schools also provided additional education or reminders about hand hygiene.

### Effectiveness of Intervention

#### Follow-up children

The rate of absence episodes due to any illness (primary outcome) was similar in the hand sanitiser (1.21 per 100 child-days) and control (1.16 per 100 child-days) groups, and the confidence interval for the IRR (IRR 1.06, 95% CI 0.94 to 1.18) excluded a clinically important difference (Table 4). Furthermore, there was no evidence that hand sanitiser was effective in reducing rates of respiratory or gastrointestinal illness episodes, or the length of the illness or illness absence episodes, to any clinically important degree. Nor did the rate of occurrence of the same illness among other members of the household subsequent to an illness in a follow-up child differ between groups. The percentage of children who had experienced a skin reaction over the period of the trial was similar between groups (10.4% hand sanitiser versus 10.3% control, OR 1.01, 95% CI 0.78 to 1.30). Pre-specified secondary analyses that adjusted for school disadvantage, in addition to city (design strata), yielded intervention effectiveness estimates that did not change appreciably compared with models that adjusted only for the design strata (Analysis S1).

**Table 4:** Estimates of effectiveness of hand sanitiser on outcome measures.

Outcome	Number Children (34 Schools per Group)		Control Group		Hand Sanitiser Group		IRR, Hand Sanitiser versus Control (95% CI)	*p*-Value	ICC* (95% CI)
	Control Group	Hand Sanitiser Group	Number of Events (Child-Days of Follow-Up)	Rate (per 100 Child-Days) or Percent	Number of Events (Child-Days of Follow-Up)	Rate (per 100 Child-Days) or Percent			
**Primary outcome**									
Number of absence episodes due to any illness‡	1,142	1,301	1,291 (111,451)	1.16	1,542 (127,471)	1.21	1.06 (0.94, 1.18)	0.346	0.018 (0.012, 0.043)
**Secondary outcomes for follow-up children**									
Number of absence episodes due to respiratory illness‡	1,142	1,301	891 (111,451)	0.80	1,069 (127,471)	0.84	1.05 (0.92, 1.20)	0.439	0.015 (0.011, 0.037)
Number of absence episodes due to gastrointestinal illness‡	1,142	1,301	159 (111,451)	0.14	196 (127,471)	0.15	1.11 (0.82, 1.52)	0.490	0.027 (0.023, 0.066)
Length of illness absence episode (total number of days child absent from school)‡	703	827	2,205 (68,786)	3.21	2,771 (80,981)	3.42	1.07 (0.96, 1.19)	0.198	0.020 (0.013, 0.051)
Length of illness episode (number of days from first to last absence day)↑	703	827	3,239 (96,302)	3.36	4,078 (113,369)	3.60	1.07 (0.96, 1.20)	0.211	0.023 (0.017, 0.061)
Number of episodes where at least one other adult in the household had the same illness after the child‡	703	826	192 (68,786)	0.28	249 (80,881)	0.31	1.08† (0.91, 1.30)	0.373	0.000†
Number of episodes where at least one other child in the household had the same illness after the child‡	703	826	226 (68,786)	0.33	301 (80,881)	0.37	1.11† (0.94, 1.30)	0.217	0.000†
**Secondary outcomes for all children (school-level analysis)** **									
Number of absence episodes for any reason	7,478	9,022	23,900 (747,800)	3.20	26,944 (902,200)	2.99	0.94 (0.84, 1.05)	0.283	
Length of absence episode for any reason	7,478	9,022	43,186 (747,800)	5.78	48,090 (902,200)	5.33	0.93 (0.81, 1.07)	0.289	
**Adverse events**									
Skin reactions	970	1,106	100	10.3%	115	10.4%	1.01† (0.78, 1.30) [OR]	0.946	0.000†

Estimates obtained from marginal models using GEEs with an exchangeable correlation structure and robust variance estimation. All models include the stratification variable “city” (Invercargill, Dunedin, or Christchurch).

*ICC point estimates are those resulting from the GEE models with no adjustment for the stratification variable. Confidence intervals for the ICCs were bootstrapped using the combination of the *bootstrap* and *xtgee* commands in Stata. Bootstrapping allowed for the clustering of observations within schools (using both the *cluster()* and *idcluster()* options). Bias-corrected 95% bootstrap confidence intervals were calculated from 5,000 replicates.

‡ The exposure period was the number of school days.

↑ The exposure period was the period the child was enrolled in the study minus the length of the school holidays.

† The ICC point estimate resulting from the GEE model for these outcomes was negative. In this circumstance, the model GEE model was refitted with an independent correlation structure, making the assumption that in the context of a cluster-based evaluation such as this, negative ICCs are more likely to occur through sampling error than because of a true negative ICC [20],[21]. Assuming an independent correlation provides more conservative estimates of the estimated standard errors.

**Data aggregated to the level of the school, and analysed at the school level. The exposure period was calculated as the average school roll over the period of the trial multiplied by 100 (the number of school days encompassed by the trial period).

#### All children

The rate of episodes of absence for any reason, and the length of episodes, calculated from absence data collected in the school rolls, did not differ importantly between the intervention and control groups (Table 4).

#### Per-protocol analysis

The per-protocol analysis, with the five control schools removed that did not comply with the protocol (i.e., introduced hand sanitiser), did not modify the intervention effectiveness for the primary outcome, rate of absence episodes due to any illness, in an important way (per-protocol intervention effectiveness estimate 1.03, 95% CI 0.93 to 1.14, *p* = 0.582).

#### Intra-cluster correlations

Estimated ICCs for all outcomes were very small (Table 4), with point estimates ranging from 0.000 to 0.027.

## Discussion

### Main Findings

We undertook a cluster randomised trial to estimate the effectiveness of hand sanitiser in reducing illness absence episodes in children in primary schools. The trial did not demonstrate that hand sanitiser reduced school absences due to illness, school absences due to respiratory or gastrointestinal illness, length of illness or of illness absence episode, or the number of times other members of the household became sick, in children followed up with post-absence interviews. In addition, skin problems were not found to be more common among children at schools where hand sanitiser was provided. The rates of absences for any reason, and of lengths of absence episodes, calculated from absence information collected in school rolls, were also similar between the intervention and control groups.

### Strengths

The trial was designed to minimise bias arising from design elements. While cluster trials are less efficient compared with individually randomised trials (although the estimated ICCs in this trial were small), this design reduces contamination that would arise if children within the same classroom or school were allocated randomly to the intervention and control groups. Furthermore, it evaluates the intervention as it would be implemented in the real world (i.e., provided to schools rather than individuals). There was adequate allocation concealment of the randomisation sequence (reducing the possibility of selection bias). Confounding by variability in viral incidence [7] was minimised through stratification by city. Outcome assessors were blind to group allocation (reducing the possibility of detection bias). Participants were not blinded to their allocation, but the use of school absences as an outcome reduces the likelihood of bias that can occur when self-reported illness is the outcome—participants' beliefs about the intervention may affect their threshold for reporting mild and non-specific symptoms such as coughs and colds, whereas caregivers' decisions to keep children at home are not likely to be so affected. A high proportion of absences were followed up with a telephone call to establish the cause of the absence, and absences that did not result in a call or where the call did not result in a record (i.e., the caregiver could not be contacted) were evenly distributed between intervention and control children (Figure 2). A further strength is the collection of absence data from school rolls, which was measured on all children and was unlikely to be affected by selection, detection, or performance biases.

### Limitations

Potential limitations of the study can be classified as issues of selection, measurement, and implementation. A higher proportion of schools in the control group were disadvantaged. We did not stratify by school disadvantage because we could not find good evidence that school disadvantage is associated with absence rates in New Zealand, so it would be unlikely to confound the effectiveness of the intervention. Pre-specified secondary analyses that adjusted for school disadvantage, in addition to city (design strata), yielded intervention effectiveness estimates that did not change appreciably compared with models that adjusted only for the design strata (Analysis S1). In fact, if school disadvantage was associated with more absences (which would be the expected direction of association, given known patterns of disease and disadvantage), it would bias the estimated intervention effect in the direction of showing greater effectiveness of hand sanitiser use than actually exists.

Another selection issue is that, as often occurs in cluster trials [23], individual participants (follow-up children) were recruited after the clusters had been randomised and the caregivers knew the allocation of the cluster. The rate of consent to be followed up after absences was not high overall (36.4%), and was lower among more disadvantaged schools. However, the effectiveness of the intervention in the analysis controlling for school disadvantage was essentially unchanged. We are unable to determine in detail whether follow-up children differed from those who did not agree to be followed up. However, despite the higher proportion of disadvantaged schools in the control group, the income and education of follow-up children's families was well balanced between the intervention and control groups, suggesting that the follow-up children in either the intervention or control group may not have been representative of their school populations. The rate of absence due to illness among controls was lower than the rate observed in data collected by the public health unit in previous years, on which our sample size calculations were based. It is possible that caregivers of follow-up children had a particular interest in hygiene and that their children were already practising good hand hygiene, and so no further benefit was possible for them. Nonetheless, the lack of effectiveness of the intervention on absences among all children suggests that our findings for illness absences are valid.

While we did not quite achieve the planned sample size of 1,350 follow-up children per group, we still obtained precise estimates of the intervention effects. This occurred because the ICCs observed in the trial (point estimates ranging from 0.000 to 0.027) were much smaller than the conservative estimate we had used in the sample size calculation (0.15).

We used telephone interviews to collect information on reasons for absence. This is less accurate than, for example, physically examining absent children, but is the only practicable way of collecting these data on the scale necessary for this study. Because our resources allowed for only a weekly visit to each school to collect data, and because of the processing time for identifying absences for follow-up children, these interviews were conducted approximately 9 d following the absences. This may have led to some inaccurate recall, but this is unlikely to have differed by group. Information on reasons for absence was incomplete, because of the failure to identify some follow-up children's absences and the inability to contact caregivers in some cases. However, these issues occurred with similar frequency in the intervention and control groups, and we do not expect the lack of a telephone call to be associated with the reason for absence, so we do not believe that these factors would have introduced meaningful bias.

Despite a pilot study that did not identify the taste of the sanitiser as a barrier to use, this did become an issue in some schools, and we changed the product in 10% of classrooms halfway through the study period. However, the quantity of sanitiser used by these classrooms following the change, which varied widely and in many cases decreased, does not support the idea that if a tasteless sanitiser had been used throughout the study, it would have been used more and would therefore have been more likely to be effective in reducing illness. The education sessions provided to intervention schools emphasised that washing hands after a number of activities is important, and that the hand sanitiser was for additional hand cleaning, reducing the likelihood of intervention children substituting hand sanitiser for use of school bathroom facilities and not actually increasing hand hygiene overall.

Performing large prospective studies of hand hygiene with reasonably long follow-up in schools is a major undertaking, and external events that may affect the purity of the design cannot be prevented [24]. The 2009 influenza A(H1N1)pdm09 pandemic began shortly after this study commenced, and by half-way through the study, there was considerable effort by government agencies to encourage the public, including schools, to practice good hand hygiene, including reminders of its importance with all media releases about the pandemic both nationally and by local health services. Poster reminders about hand hygiene, and in some cases hand sanitiser, were provided in many places such as public venues, shopping centres, and hospitals. Most schools in this study responded by encouraging better hand hygiene and social distancing via school newsletters and reminders to children, and some control schools installed hand sanitiser (we do not have information for children in the study about the individual use of hand sanitiser provided by caregivers). While having contemporaneous controls should deal appropriately with changes that occur over time that are unrelated to the intervention (and this provides a good example of why controlled trials are necessary), there are two potential consequences of the schools' actions that may have affected the intervention effectiveness. First, although children in intervention schools could be argued to have had better opportunities than control children to respond to hand hygiene messaging by using sanitiser, the more intensive hand hygiene education that may have ensued as a result of the pandemic may have increased hand hygiene behaviours in all schools, decreasing the measurable impact of the intervention. Second, the introduction of hand sanitisers in some control schools introduced contamination across groups, potentially biasing the intervention effect. However, the per-protocol analysis did not modify the intervention effectiveness for the primary outcome in an important way.

### Generalisability

The participation rate among eligible schools (50%) was not high; however, in a randomised controlled trial this is an issue of generalisability (external validity) rather than bias in the measured intervention effect (internal validity). Participating schools tended to be smaller, but all had at least 100 pupils, and it seems unlikely that the effectiveness of hand hygiene would differ depending on school size within this range, suggesting that our findings are generalisable to primary school children in high-income countries.

### These Results in the Context of Other Studies

A systematic review and meta-analysis published by Aiello et al. in 2008 included a number of studies of the effect of hand hygiene interventions on gastrointestinal, respiratory, and combined illnesses in community settings, many of which were schools [5]. They report a rate ratio of “combined illnesses” of 0.74 (95% CI 0.59 to 0.93) for interventions involving alcohol-based hand sanitiser compared with control, based on two studies [11],[25]. Both these studies were set in primary schools, and both were funded by manufacturers of hand sanitiser. One appears to be a non-randomised comparison of sanitiser provision with usual practice [11]; the other was a cross-over study that compared an education session with sanitiser provision plus an enhanced education session, undertaken in a single school with 5 wk of follow-up in each intervention period [25]. Aiello et al. note in their review that studies that did not randomise and those with shorter follow-up found stronger effects on combined illnesses, and this may partly explain why our results differ from those of these two previous studies. Aiello et al. identified no studies that compared intervention with an alcohol-based hand sanitiser with control and measured respiratory or gastrointestinal illnesses separately. However, there were a number of studies that compared provision of alcohol-based hand sanitiser plus hand hygiene education with a control group (this is not the same as our study since we provided hand hygiene education to both the intervention and control groups). For these studies, rate ratios for the intervention group were 0.77 (95% CI 0.52 to 1.13; five studies) for gastrointestinal illness, 0.93 (95% CI 0.84 to 1.03; six studies) for respiratory illness, and 0.79 (95% CI 0.67 to 0.93; three studies) for combined illnesses. Since this systematic review, the results of two cluster randomised controlled trials conducted in primary schools in the US have been reported [9],[13]. One, a trial that randomised ten schools, found a reduction in laboratory-confirmed influenza A infections in the intervention group, but there was no difference in the study's pre-specified primary outcome of all influenza infections (adjusted IRR 0.81, 95% CI 0.54 to 1.23) [9]. The other, which randomised six “teams” of classes either to an intervention that included hand sanitiser and daily surface cleaning or to usual practice, and followed up for 8 wk, found a reduction in school absences due to gastrointestinal illness (adjusted IRR 0.91, 95% CI 0.87 to 0.94) but not absences due to respiratory illness (adjusted IRR 1.10, 95% CI 0.97 to 1.24) [13].

### Implications

This study shows that adding hand sanitiser to usual school hand hygiene practices in New Zealand does not prevent disease of a severity to warrant school absence. In particular, we found that during an influenza pandemic [26],[27], when any impact of hand sanitiser would be particularly important, providing hand sanitiser was not an effective mechanism for reducing illness absence. Our study does not address the effectiveness of hand sanitiser for reducing specific infections such as influenza, and we have not shown that hand hygiene itself is not important, nor that hand sanitiser as a method of hand hygiene is not useful. Where clean water is scarce, hand sanitiser could be a useful alternative [28]. However, our results suggest that in a high-income country, putting resources into extra hand hygiene by providing hand sanitiser in classrooms may not be effective in reducing illness absences.

We undertook this trial because school absence due to illness is common in New Zealand, and an intervention that could halve it, as some trials suggested at the time we designed the study, or even reduce it by 25%, as suggested by the meta-analysis by Aiello et al. [5], would be important and useful. However, good-quality and more recent studies in schools in high-income countries, including ours, show that the addition of hand sanitiser to existing hand hygiene facilities does not result in important benefits. An updated systematic review of the impact of different hand hygiene interventions for reducing school absence in high-income countries should be a high priority before further such trials are carried out.

## Supporting Information

Protocol S1 **Trial protocol.**
(PDF)

Checklist S1 **CONSORT Statement checklist.**
(DOCX)

Deviations S1 **Protocol deviations.**
(DOCX)

Analysis S1 **Analysis adjusting for deprivation.**
(DOCX)

## Abbreviations

GEE: generalised estimating equation
ICC: intra-cluster correlation
IRR: incidence rate ratio
OR: odds ratio
