# Supplementary material for: Hand Sanitiser Provision for Reducing Illness Absences in Primary School Children: A Cluster Randomised Trial
Source: PLoS Med. 2014 Aug 12;11(8):e1001700. doi: 10.1371/journal.pmed.1001700 (PMC4130492; doi:10.1371/journal.pmed.1001700)
Supplement: Deviations S1 — Protocol deviations. (DOCX) [file pmed.1001700.s003.docx]

Hand sanitiser provision for reducing illness absences in primary school children: Cluster randomised trial in New Zealand

**PROTOCOL DEVIATIONS – DETAIL**

Some changes were made to the protocol following the protocol’s publication.[[1](#_ENREF_1)] – all were made prior to the commencement of analysis.

*Measures*

*Length of illness episode* (defined as the number of days from the first day to the last day of an absence episode): the protocol stated that children who were absent only on a Monday or only on a Friday would be considered to have an illness lasting 2 days, to account for the likelihood that they had been sick for one of the weekend days as well. However we realised that this should also apply to absences lasting more than one day but abutting a weekend day. Therefore, in the analysis that was carried out the length of illness episode was calculated to be the number of days from the first to last day of the absence episode, plus one day if the first day of the absence episode was a Monday and plus one day if the last day of the absence episode was a Friday. Thus a child who was absent from Monday to Wednesday was considered to have an **illness** episode of 4 days; if the absence episode ran from Monday to Friday then the illness episode would be 7 days.

*Reason for absence*: The protocol (pg 7, col 2, para 1) stated that where we could not contact a follow-up child’s parents after an absence, we may be able to determine the reason for absence from the school administrative staff. However, we found that information about the reason for absence was very variably recorded by schools, so it was decided that information noted on the school rolls would not be used in the analysis of the follow-up children.

*Definitions of respiratory and gastrointestinal illness:* The definitions stated in the protocol were based on those used in a previous trial[[2](#_ENREF_2)] of hand sanitiser in schools:

“A respiratory illness will be defined as an acute illness that includes at least one of the following symptoms: runny nose, stuffy or blocked nose, cough, fever or chills, sore throat, or sneezing. A gastrointestinal illness will be defined as an acute illness that includes at least two watery or much looser than normal bowel movements and stools over a 24 hour period and/or vomiting.”[[1](#_ENREF_1)]

However, this would define an illness with fever as the only symptom as a respiratory illness, which on reflection did not seem reasonable. In addition, because of the time between the symptoms and the telephone interview, our questionnaire had been amended to ask about ‘diarrhoea’ rather than specifying a number of watery stools over a 24 hour period. We also needed to make a decision about how to classify children who had had both respiratory and gastrointestinal symptoms, since vomiting in particular can be a feature of febrile respiratory illnesses.

The group who performed the trial we based our definition on have done several studies of the effect of hand sanitiser on illness and had initially used definitions of respiratory and gastrointestinal illness generated by an expert panel:[[3](#_ENREF_3)]

A respiratory illness was defined as 2 of the following symptoms for 1 day or 1 of the following symptoms for 2 consecutive days, not including 2 consecutive days of cough alone, sneezing alone, or fever alone: (1) runny nose, (2) stuffy or blocked nose or noisy breathing, (3) cough, (4) feeling hot or feverish or having chills, (5) sore throat, or (6) sneezing. A new or separate respiratory illness was defined as 1 of the following: (1) the occurrence of a respiratory illness (as defined above) after a period of 3 illness-free days or (2) if the caregiver stated that the child or other household member had a new illness. A GI illness in adults and children >6 years was defined as 1 or both of the following on the basis of our expert panel and literature review: (1) any episode of watery or much looser than normal bowel movements or stools or (2) vomiting. In children 0 to 5 years of age, a GI illness was defined as 1 or both of the following: (1) 2 or more watery or much looser than normal bowel movements or stools over a 24-hour period or (2) any episode of vomiting or forceful expulsion of stomach contents, not including spit-ups for infants or young children. New or separate GI illnesses were defined as 1 of the following: (1) the occurrence of a GI illness (as defined above) after a period of 3 illness-free days or (2) if the caregiver stated that the household member had a new illness.[[3](#_ENREF_3)]

We decided to adapt the definitions we had planned to use, to prevent fever alone being labelled ‘respiratory disease’ and to be more consistent with the definitions proposed by the earlier expert panel. The definitions actually used in the analysis were therefore:

Respiratory illness: An episode of illness that includes at least 2 of the following caregiver-reported symptoms for 1 day or 1 of these symptoms for 2 days **(but not fever alone)**: (1) runny nose; (2) stuffy or blocked nose or noisy breathing; (3) cough; (4) fever; (5) sore throat; and (6) sneezing.

Gastrointestinal illness: An episode of illness that does not meet the criteria for respiratory illness and includes either or both of the following symptoms lasting for at least 1 day: (1) diarrhoea and (2) vomiting.

*Definition of per protocol analysis*

In the protocol the per-protocol analysis is stated as being:

“A secondary per-protocol analysis will be undertaken where we will only include schools which complied with their allocated intervention. For the intervention group, we will define schools as complying if they used at least 45 ml per child of hand sanitiser solution over the study period. This usage equates to using the hand sanitiser at least once per day.”

We had not anticipated that control schools would install hand sanitiser, because the inclusion criteria for the study required that they agree not to. However, in view of the pandemic of Influenza A (H1N1)pdm09 during the study period, some control schools did install hand sanitiser in response to public health advice to increase hand cleaning. Therefore, the per-protocol analysis defines compliance for intervention schools as above, and in addition defines compliance for control schools as:

“For the control group, we will define schools as complying if they did not install hand sanitisers for use by students at any time throughout the trial.”

1. McKenzie J, Priest P, Audas R, Poore M, Brunton C, Reeves L. Hand sanitisers for reducing illness absences in primary school children in New Zealand: a cluster randomised controlled trial study protocol. Trials 2010;**11**(1):7

2. Sandora TJ, Shih MC, Goldmann DA. Reducing absenteeism from gastrointestinal and respiratory illness in elementary school students: a randomized, controlled trial of an infection-control intervention. Pediatrics 2008;**121**(6):e1555-62

3. Lee GM, Salomon JA, Friedman JF, et al. Illness transmission in the home: a possible role for alcohol-based hand gels. Pediatrics 2005;**115**(4):852-60
