# Supplementary material for: Hand Sanitiser Provision for Reducing Illness Absences in Primary School Children: A Cluster Randomised Trial
Source: PLoS Med. 2014 Aug 12;11(8):e1001700. doi: 10.1371/journal.pmed.1001700 (PMC4130492; doi:10.1371/journal.pmed.1001700)
Supplement: Analysis S1 — Analysis adjusting for deprivation. (DOCX) [file pmed.1001700.s004.docx]

**Table: Effect of hand sanitisers on outcome measures (adjustment for design strata and school level deprivation)**

| **Outcome** | **Number Children (34 Schools per Group)** | | **Control Group** | | **Hand Sanitiser Group** | | **IRR, Hand Sanitiser versus Control (95% CI)** | ***p*-Value** | |
| --- | --- | --- | --- | --- | --- | --- | --- | --- | --- |
|  | **Control Group** | **Hand Sanitiser Group** | **Number of Events (Child-Days of Follow-Up)** | **Rate (per 100 Child-Days) or Percent** | **Number of Events (Child-Days of Follow-Up)** | **Rate (per 100 Child-Days) or Percent** |  |  |  |
| **Primary outcome** |  |  |  |  |  |  |  |  | |
| Number of absence episodes due to any illness‡ | 1,142 | 1,301 | 1,291 (111,451) | 1.16 | 1,542 (127,471) | 1.21 | 1.07 (0.96, 1.19) | | 0.223 |
| **Secondary outcomes for follow-up children** |  |  |  |  |  |  |  | |  |
| Number of absence episodes due to respiratory illness‡ | 1,142 | 1,301 | 891 (111,451) | 0.80 | 1,069 (127,471) | 0.84 | 1.07 (0.94, 1.22) | | 0.296 |
| Number of absence episodes due to gastrointestinal illness‡ | 1,142 | 1,301 | 159 (111,451) | 0.14 | 196 (127,471) | 0.15 | 1.14 (0.83, 1.55) | | 0.422 |
| Length of illness absence episode  (total number of days child absent from school) ‡ | 703 | 827 | 2,205 (68,786) | 3.21 | 2,771 (80,981) | 3.42 | 1.09 (0.98, 1.21) | | 0.103 |
| Length of illness episode  (number of days from first to last absence day)↑ | 703 | 827 | 3,239 (96,302) | 3.36 | 4,078 (113,369) | 3.60 | 1.09 (0.98, 1.21) | | 0.101 |
| Number of episodes where at least one other adult in the household had the same illness after the child ‡ | 703 | 826 | 192 (68,786) | 0.28 | 249 (80,881) | 0.31 | 1.1^†^ (0.94, 1.31) | | 0.228 |
| Number of episodes where at least one other child in the household had the same illness after the child ‡ | 703 | 826 | 226 (68,786) | 0.33 | 301 (80,881) | 0.37 | 1.12^†^ (0.95, 1.32) | | 0.172 |
| **Secondary outcomes for all children (school-level analysis)**** |  |  |  |  |  |  |  | |  |
| Number of absence episodes for any reason | 7,478 | 9,022 | 23,900 (747,800) | 3.20 | 26,944 (902,200) | 2.99 | 0.98 (0.91, 1.05) | | 0.558 |
| Length of absence episode | 7,478 | 9,022 | 43,186 (747,800) | 5.78 | 48,090 (902,200) | 5.33 | 0.97 (0.88, 1.07) | | 0.522 |
| **Adverse events** |  |  |  |  |  |  |  | |  |
| Skin reactions | 970 | 1,106 | 100 | 10.3% | 115 | 10.4% | [OR] 1.03^†^ (0.81, 1.31) | | 0.798 |

Estimates obtained from marginal models using generalised estimating equations (GEE) with an exchangeable correlation structure and robust variance estimation. All models include the stratification variable “region” (Invercargill, Dunedin, Christchurch) and school level deprivation. School level deprivation is measured using a decile assigned to each school by the Ministry of Education for funding purposes.

† The ICC point estimate resulting from the GEE model for these outcomes was negative. In this circumstance, the model GEE model was refitted with an independent correlation structure, making the assumption that the in the context of a cluster based evaluation such as this, negative ICCs are more likely to occur through sampling error than because of a true negative ICC (Ukoumunne OC, Gulliford MC, Chinn S, Sterne JA, Burney PG. Methods for evaluating area-wide and organisation-based interventions in health and health care: a systematic review. Health Technol Assess. 1999;3(5):iii-92; Eldridge SM, Ukoumunne OC, Carlin JB. The intra-cluster correlation coefficient in cluster ranomized trials: A review of definitions. International Statistical Review / Revue Internationale de Statistique. 2009;77(3):378-94.). Assuming an independent correlation provides more conservative estimates of the estimated standard errors.

‡ The exposure period was the number of school days.

↑ The exposure period was the period the child was enrolled in the study minus the length of the school holidays.

** Data aggregated to the level of the school, and analysed at the school level. The exposure period was calculated as the average school roll over the period of the trial multiplied by 100 (the number of school days which were encompassed by the trial period).
